# Ultra-Brief Crisis IPT-A Based Intervention for Suicidal Children and Adolescents (IPT-A-SCI) Pilot Study Results

**DOI:** 10.3389/fpsyt.2020.553422

**Published:** 2020-12-09

**Authors:** Liat Haruvi Catalan, Mira Levis Frenk, Ella Adini Spigelman, Yair Engelberg, Shira Barzilay, Laura Mufson, Alan Apter, Noa Benaroya Milshtein, Silvana Fennig, Anat Brunstein Klomek

**Affiliations:** ^1^Schneider Children's Medical Center of Israel, Petach Tikva, Israel; ^2^Department of Psychiatry, Columbia University and New York State Psychiatric Institute, New York, NY, United States; ^3^Interdisciplinary Center (IDC), School of Psychology, Herzlyia, Israel; ^4^Ruppin Academic Center, Emek Hefer, Israel; ^5^Sackler Medical School, Tel Aviv University, Tel Aviv, Israel

**Keywords:** suicide attemps, crisis intervention, depression, adolescents, suicide behavior, IPT

## Abstract

In recent years, suicidal behaviors have shown substantial increase worldwide. This trend is also prominent in Israel and has led to a dramatic increase in mental health treatment demand resulting in long wait times and low treatment acceptance rate. To address the critical need in crisis intervention for children and adolescents at suicidal risk we developed an ultra-brief acute crisis intervention, based on Interpersonal Psychotherapy (IPT). IPT is an evidence-based intervention for various psychopathologies among different age groups. The current adaptation of IPT-A is comprised of five weekly sessions, followed by monthly follow-up caring email contacts to the patients and their parents, over a period of 3 months. This paper aims to review the theoretical foundation of this intervention, describe the research design, and present preliminary results of a pilot study. Preliminary Results from our samples of 26 adolescents indicate meaningful trends for both the suicidal ideation (SIQ) and depression (MFQ) outcome measures. Significant interaction was found concerning suicidal ideation but not for depression. Main limitations include small sample size and stratified controls. The treatment appears to be safe, feasible and acceptable and initial results show promising trends to support further study of the approach.

## Introduction

Suicidal ideation and behavior are major public health concerns. Data in recent years indicates an alarming increase in the prevalence of suicide attempts, particularly among adolescents (1). Although only a small proportion of suicide attempts are fatal, every attempt is fraught with a potential death and long-term physical and psychological effects (2). Suicidal ideation and non-suicidal self-injurious (NSSI) behaviors have been identified as precursors for suicide attempts as well as potent risk factors for eventual death. Despite the unequivocal need for immediate and focused interventions for teens presenting with suicidal ideation, NSSI, and suicidal behavior, to our knowledge, there are few evidence-based, suicide specific, interventions for these at-risk youth. Dialectical Behavioral Therapy for Adolescents (DBT-A) for example has good evidence-base and dissemination (3–5). Other evidenced based psychotherapies include Cognitive Behavioral Therapy (CBT) (6), Mentalization Based Therapy (MBT) (7) and Attachment – Based Family Therapy (ABFT) (8).

In recent years these non-fatal suicidal behaviors have shown substantial increase despite trends in the Western world for a reduction in actual suicide rates. This is apart from the US where adolescent suicide rates have climbed probably due to the epidemic in opioid abuse (9). This increase in non-fatal suicide behavior has been seen in Israel as well (10).

The rate of presentations to the pediatric emergency room (ER) at Schneider Children's Medical Center (SCMC) for suicidal behavior (i.e., sever ideation, suicide attempts, NSSI) increased in recent years and now stands at about 400 cases per year. This increase in pediatric admissions to the ER has led to an overload on outpatient services for our adolescent depression and suicidal behavior clinic.

These circumstances, have led to a long average wait time (at least 1 year) and, low treatment acceptance rate (<10%), which is clinically unreasonable when it comes to children and youth suffering from severe depression and suicidal risk. In order to meet these needs and deal with this public health crisis, we have developed an ultra-brief acute crisis intervention consisting of five sessions which we are currently evaluating in an ongoing study. This paper aims to present the theoretical basis of this ultra-brief intervention, describe the research design, and present some preliminary results from a pilot study.

The intervention developed is based on Interpersonal Psychotherapy (IPT) and on a comparable short-term intervention for suicidal adults (11).

The selection of IPT is based on the many studies linking between interpersonal problems and suicide risk (12). Those at risk for suicide suffer from significant interpersonal challenges (13). Insecure attachment has specifically been found to be a risk factor for suicide in adolescents (14). Moreover, problems with sharing feelings with others have been found to be an important risk factor for severe suicide attempts, above and beyond the contribution of depression and hopelessness (15, 16).

IPT is a commonly used and evidence-based treatment for depression among adults (17, 18) and adolescents [IPT-A, (19, 20)] as well as for several other disorders such as anxiety (21) and eating disorders (22). It has been shown to be effective in reducing symptoms of depression and in improving overall performance and social functioning of patients (23, 24).

IPT-A, is an adaptation of the IPT protocol specifically tailored for depressed adolescents (19). It is a time-limited evidence-based psychotherapy that addresses the link between depressed mood and current interpersonal problems. The goal of IPT-A is to reduce depressive symptoms and improve interpersonal functioning by identifying an interpersonal problem area of focus and by developing communication and problem-solving strategies for dealing with the interpersonal problems (19). Mufson et al. (25) presented preliminary outcomes of a small sample of IPT-A for depressed youth engaging in suicidal behavior (IPT-A-SP). The results indicated that IPT-A is a feasible treatment for adolescents at risk for suicide. Graham et al. (26) are in the process of publishing their work on an adaptation of the IPT protocol for suicidal adults as a part of their crisis intervention treatment (**IPT-AC)**. To our knowledge, no other IPT-based adaptation suitable for suicidal youth is available or has been empirically examined.

### Short Term Intervention in Suicidal Adults

Studies carried out among adults have shown that brief and focused therapy can be effective for patients at high risk of suicide. For example, Gysin-Maillart et al. (11) compared two groups of adults presenting to the ER following a suicide attempt. Both groups received treatment as usual (TAU), which included inpatient, day patient, and individual outpatient care as considered necessary by the clinicians in charge of patient management. The research group received, in addition to TAU, three 60–90-min therapy sessions that were focused on the suicide attempt. This group of patients also received personal letters from the clinic every 3–6 months for a period of 24 months. The results showed a significant decrease in suicide attempts within the research condition in comparison to the control group.

Another study followed 843 adults who were hospitalized due to depression or suicidal risk in a psychiatric ward in San Francisco and refused further treatment upon their release. The study was conducted over the course of 5 years, comparing two groups of participants; The experimental group, which received personal postcards 4 times per year for the duration of the study, and the control group which received no further contact. A follow-up procedure identified patients who died during the 5-year contact period. Suicide rates in the contact (experimental) and no-contact groups were compared. The 5-year-follow-up revealed lower suicide rates within the contact group compared to the control group (27).

Based on these two lines of studies (IPT-A and short-term interventions for suicidal adults) we set out to develop and examine a brief and focused IPT-A based acute crisis intervention, adapted for suicidal children and adolescents. To the best of our knowledge, despite the tremendous service gap and devastating consequences of the global suicide epidemic among children and adolescents, there is no study which examines a very short, practical and feasible crisis intervention for children and adolescents at risk of suicide.

### The Intervention

The intervention has two main goals: (1) **Immediate intervention for depression and suicide ideation**. We offer a very short and focused treatment, allowing for immediate response in cases of suicidal risk. By being ultra-short, the protocol may be offered to more youth within a significantly shorter wait time; (2) **Building a roadmap for future treatment after the suicidal risk is reduced**. During the intervention, we build a roadmap for continued patient care in the community. We try to identify the main difficulties leading to the patient's suicidal behavior, and to assist the patient and family in understanding how these needs can be met.

The intervention is comprised of five weekly sessions followed by monthly emails to the patients and their parents over a period of 3 months. The first session is aimed at introducing the intervention, assessing depression and suicidal risk and building a safety plan (28). The safety plan consists of a prioritized list of coping strategies that the patient can use when suicidal risk is increased. The second session is focused on reviewing the patient's interpersonal relationships (using the closeness circle and interpersonal inventory) and conceptualizing the interpersonal problem area (the focus for the intervention). Sessions 3–4 focus on developing and practicing interpersonal, emotional and behavioral coping strategies relevant for suicidal risk. Lastly, in session 5, patient and therapist go over the process and main issues which were worked on, emphasizing relapse prevention by going back to the safety plan. First and fifth sessions always involve the parents. In other sessions, parents are invited as needed. After completion of the intervention, four personal emails addressed to the patients and parents are sent by the clinic. These are sent two, four, eight, and 12 weeks following the last session. The email messages are based on a template that includes care contacts, elements of the safety plan and skills acquired during the intervention, suggestions to parents and patients and emergency contact information.

### Research Design

The goal of the study is to examine the feasibility and effectiveness of an ultra-brief IPT-A based acute crisis-intervention, as first aid for suicidal children and adolescents, in an outpatient setting. The study was approved by IRB committee at Schneider Children's Medical Center (SCMC). The study was registered as clinical trial in the national institution of mental health ref. no: NCT04404322

## Methods

### Participants

All study participants are patients at the SCMC depression and suicidal behavior clinic, referred due to depressive symptoms and/or suicidal ideation/behavior. Patients receive routine care and those who give informed assent and guardian consent are assessed routinely via a battery of self and parent report questionnaires. All study participants and their parents signed informed consent forms.

Since the beginning of the study about 80 children and adolescents, aged 6–18, have consented to fill study baseline and follow up assessments. Exclusion criteria include acute medical condition, intellectual disability, cognitive impairment, or linguistic limitation. We present here preliminary data of the first 26 children/adolescents recruited who have completed 2–3 measurement timepoints (Initial evaluation, Pre-treatment, Post-treatment). The sample included 10 males and 16 females, between the ages of 9–17 (Mean = 13.44, SD = 2.45).

### Procedure

Patients are referred to SCMC depression and suicide clinic through the ER, outpatient providers or are self-referred. They undergo an initial evaluation and risk assessment. Following initial evaluation, each subject is assigned to one of three study groups, based on clinical considerations. In this stratified randomization system, the most serious acute cases are generally referred to our ultra-short crisis intervention (IPT- A SCI) and the rest are randomized to either one of the three groups: IPT- A SCI, Treatment as usual (TAU) and waiting list (WL). All participants and their parents complete the questionnaires via a secure electronic interface, with the aid of a trained research assist ant. Post treatment drop-out rates, thus far, seem low and stand at about 11% for the IPT-A SCI and at about 16% for patients who receive TAU.

The IPT-A SCI follows the intervention protocol briefly presented above, which includes an intensive phase of 5 weekly 50-min sessions and 3 follow up personal emails. Questionnaires are completed at baseline (initial evaluation), prior to the first session; at the end of the intensive phase; and 3, 6, 9, and 12 months following the end of the acute intervention. TAU patients receive an integrative combination of psychodynamic, supportive and cognitive behavioral therapy, usually lasting between 10 and 30 weeks. TAU participants are assessed at the same time points with an added measurement at the end of treatment. WL patients are monitored by a trained clinician during their waiting period and complete the study questionnaire battery at the parallel time intervals. Table 1 summarizes the measurement timeline for each study group.

**Table 1:** Measurement timeline for each study group.

**Time**	**IPT-A-SCI**	**TAU**	**WL**
0	Initial evaluation	Initial evaluation	Initial evaluation
1	Pre-treatment	Pre-treatment	–
2	Post-treatment	5-week assessment	5-week assessment
3	–	Post treatment	–
4	3-months follow up (from the end of the intervention)	3-months follow up (from the end of the intervention)	3-months follow up (from the end of last evaluation)
5	6 months follow up	6 months follow up	6 months follow up
6	9 months follow up	9 months follow up	9 months follow up
7	12 months follow up	12 months follow up	12 months follow up

### Instruments

The study included a battery of validated self- and parent-report questionnaires assessing suicidal ideation [Suicide Ideation Questionnaire (SIQ) (29)] and behavior [Columbia–Suicide Severity Rating Scale (C-SSRS) (30)], interpersonal functioning [Social Adjustment Scale–Self Report (SAS-SR) (31)], depressive symptoms [Mood and Feeling Questionnaire (MFQ) (32)], anxiety [The Screen for Child Anxiety Related Emotional Disorder (SCARED) (33)], attachment patterns [Experiences In Close Relationships – Revised child version Questionnaire (ECR-RC) (34)] and self-esteem [Rosenberg Self-esteem Scale (RSE) (35)]. In this report we will only discuss the SIQ and MFQ, which are the two primary outcome measures initially identified for the study. The SIQ consists 15-item measure designed to evaluate the severity and frequency of suicidal ideation. Scale ranges from 1 (“I never had this thought”) to 7 (“Almost every day”) according to their thoughts in the last month. The MFQ consists of a series of 13 descriptive phrases regarding how the subject has been feeling or acting recently, assessing depressive symptoms of children and youth.

## Results

In order to estimate the feasibility of this intervention, analyses were conducted on the first 26 children/adolescents who completed between 2 and 3 assessment timepoints. Initial analysis of group differences at baseline showed no differences in demographic (age and gender) characteristics or in outcome measures between the study arms. A repeated measures design was used to compare three groups of patients who completed the SIQ and MFQ at initial evaluation (T0), pre-intervention (T1) and at 5-week assessment (T2). Ten WL patients completed the initial evaluation and the 5-week follow up assessments, 10 IPT A SCI patients completed initial evaluation, pre-intervention and post-intervention assessments and 6 TAU patients completed initial evaluation, pre-intervention and 5-week follow up assessments.

Results are meaningful for both the SIQ and MFQ outcome measures. Specifically, a significant interaction was evident for suicidal ideation (F_(4, 46)_ = 3.34, *p* < 0.05); it appears that while WL patients exhibited a slight increase in their suicidal ideation levels between T0 and T2 (t_(9)_ = −2.75, *p* < 0.05), suicidal ideation decreased in the IPT-A SCI and TAU patients, between both time intervals. Paired comparisons within treatment condition using Bonferroni adjustment revealed significant differences between T0 and T2 measurements for the IPT-A SCI group (*p* < 0.05) but not for TAU. Means and standard deviations are presented in Table 2.

**Table 2:** Means and Standard deviations of each group at different times.

**Outcome**	**Group**	**T0**	**T1**	**T2**	**Sig**
		**Mean (SD)**	**Mean (SD)**	**Mean (SD)**	**differences**
SIQ	IPT-A SCI	57.4 (24.7)	29.4 (24.8)	17.6 (18.3)	**T0** **>** T2^*^
	TAU	62.5 (27.8)	37.6 (35.8)	21.5 (34.54)	
	WL	57.7 (14.98)	–	68.2 (14.24)	**T0** **<** T2^*^
MFQ	IPT-A SCI	83.5 (31.3)	20.3 (17.7)	15.7 (15.08)	**T0** **>** T1^**^ **T0** **>** T2^**^
	TAU	89.5 (29.43)	28.33 (20.44)	19.33 (21.67)	**T0** **>** T1^**^ **T0** **>** T2^**^
	WL	76.0 (24.99)	–	15.5 (13.2)	**T0** **>** T2^**^

* *p <0.05*.

** *p < 0.001*.

**Figure 1:**
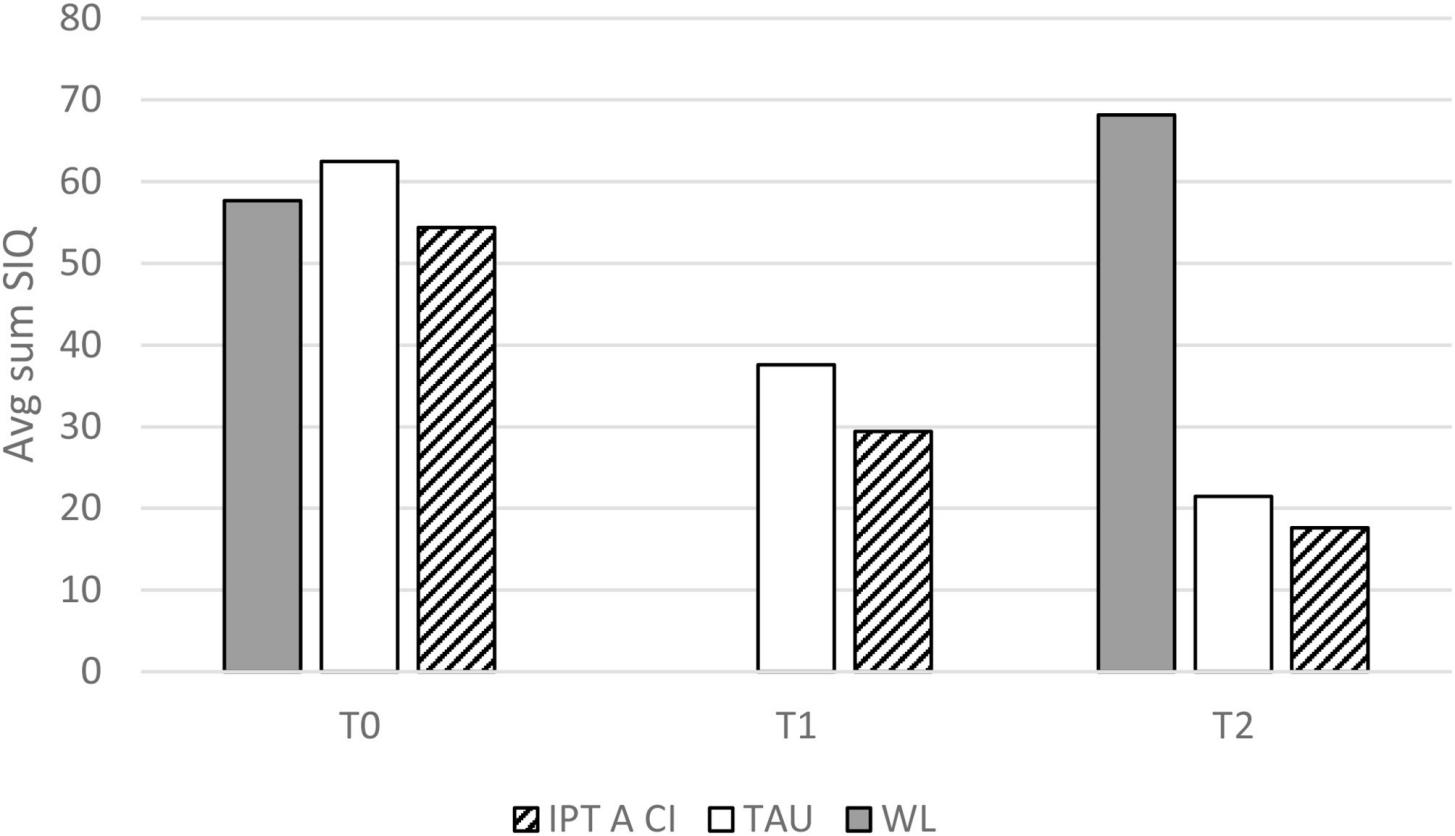
Suicidal ideation by group and measurement.

**Figure 2:**
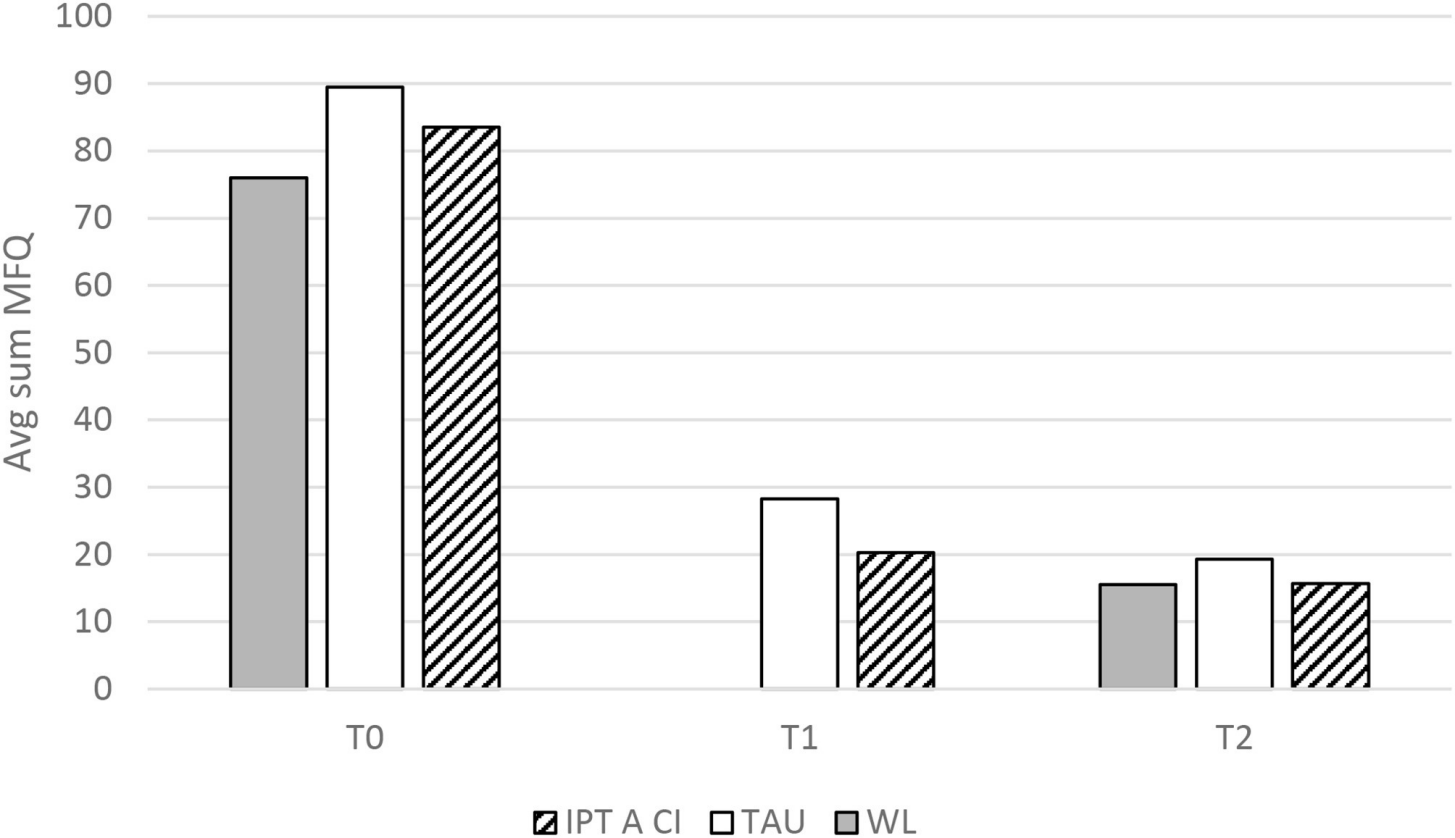
Depressive symptomology by group and measurement.

For depressive symptoms however, no interaction effect was evident and all three patient groups exhibited a reduction across timepoints. Means and standard deviations are presented in Table 2. Paired comparisons using Bonferroni adjustment revealed significant differences between T0 and T2 for all patients (*P* < 0.001), as well as significant reductions between T0 and T1 for the TAU and IPT-A SCI groups (*P* < 0.001). Nevertheless, the slope seems to flatten between T1 and T2, and these very preliminary results might suggest that the level of depression may not be specifically affected by the initiation of any of the interventions. Figures 1, 2 illustrate the above findings.

## Discussion

In this brief report, we have described a new adaptation of an acute ultra-brief crisis intervention for children and adolescents at suicidal risk, developed in our depression and suicide clinic at SCMC, and reviewed its theoretical foundations. It seems that the intervention is feasible and safe. The intervention group was superior to wait list group in reducing suicide ideation. The intervention, however, did not differ from the two comparison groups in reducing symptoms of depression. The difference in outcomes for the suicide ideation and depression needs further investigation. We could assume that this is due to the suicide-specific nature of the intervention protocol we present.

The treatment appeared to be acceptable to subjects and parents, and, at least in this pilot stage, dropout rates were very low, which is encouraging, since treatment studies of suicidal adolescents are notorious for high numbers of dropout (36).

IPT-A is a relevant intervention for at risk youth since it focuses on the interpersonal aspects which often precipitate acute suicidal crises. All available interventions [CBT (6) DBT (4)] include interpersonal aspects but these aspects are not necessarily the focus of the treatment. ABFT focuses on interpersonal aspects in a family format (8).

IPT frames therapy around a main interpersonal problem in the patient's life, a crisis or relational issue that is increasing interpersonal stress. According to (37) by mobilizing and working collaboratively with the patient to resolve this problem, IPT seeks to activate several interpersonal change mechanisms. These include: enhancing social support; decreasing interpersonal stress; facilitating emotional processing; and improving interpersonal skills.

None of these change mechanisms are unique to IPT, and not always all factors have the same importance in every treatment. However, IPT's uniqueness lies in seeking to activate all of these in a coherent, plausible therapeutic frame, defined by a current interpersonal crisis or predicament in the patient's lifeand in a time-limited, diagnosis-focused treatment (37).

Until recently, many studies that have examined the efficacy of IPT-A have generally excluded participants with severe and active suicidal ideation and attempts despite the possibility that IPT-A may be beneficial for this population.

We believe that IPT-based intervention can be particularly suitable for children and adolescents at risk of suicide for a number of reasons. First, the IPT framework makes it possible to focus the suicidal ideation or behavior around a specific problem area or difficulty that especially affects the patient. Second, the work in the interpersonal context is very suitable for young patients at suicide risk. In the intervention this work is reflected in the construction of a safety plan and in increasing the patient's support system. Lastly, the protocol is focused and limited in time so that it can be adapted relatively easily to the model of emergency intervention

The intervention presented in the current study is the first to focus on interpersonal aspects in an ultra-short individual format. It is not our supposition that this treatment is sufficient for these adolescents, but it may be a cost-effective “first aid” intervention, which will help them through an acute crisis as well as generate hope regarding the prospect of therapy, thereby getting them to engage in longer term services.

Using this ultra-brief intervention has important public health implications. Non-fatal adolescent suicidal behavior, in all of its various forms, has become increasingly common in the last decades. These high-risk behaviors pose a major public health problem, imposing unbearable load on emergency rooms and public psychiatric services. Public mental health services in Israel struggle to handle the load, cases may be left un-treated for dangerously long periods of time. Therefore, there is a real need a for brief forms of crises interventions. Intervention like IPT-A-SCI may be able to reduce the load on child and adolescent mental health services and allow larger portions of at-risk patients to be treated. Unfortunately, we are not able to show a significant decrease in waiting time for treatment since the number of referrals is still increasing. However, we were able to increase the number of children receiving treatment by a significant number. Before the initiation of the treatment program we were providing psychotherapy to 30–40 patients per year, while during the study period we are able to treat 80–100 patients per year – a very significant increase.

Furthermore, provided our future large-scale study's results continue the promising trends evident in our pilot analyses, this protocol can be a basis for other types of crises interventions in various fields and populations. Likewise, our protocol could potentially be adapted to other populations at other age groups or suffering from other psychopathologies. In addition, IPT-A-SCI could serve as a platform for crises interventions based on other therapeutic models such as CBT or DBT, whereby a 12–16 session long protocol is condensed into a 5-session “first aid kit,” maintaining the main therapeutic components and directing the patient for future care.

This study does have limitations. Firstly, the treatment sample was relatively small and in addition we only had limited number of assessments over the treatment period. We also did not have enough quality control for the individual's sessions. Finally, due to the nature of our suicidal adolescents who needed urgent treatment, we could only use stratified controls and had to give priority to the more urgent cases.

Despite the many limitations of this preliminary report, the treatment has proven safe, feasible and acceptable and initial results show promising trends. We are in the process of developing a larger, randomized control study, which will include a larger sample and the other measures included.

## Data Availability Statement

The raw data supporting the conclusions of this article will be made available by the authors, without undue reservation.

## Ethics Statement

The studies involving human participants were reviewed and approved by IRB Helsinki Committee, Rabin Medical Center. Written informed consent to participate in this study was provided by the participants' legal guardian/next of kin.

## Author Contributions

All authors listed have made a substantial, direct and intellectual contribution to the work, and approved it for publication.

## Conflict of Interest

The authors declare that the research was conducted in the absence of any commercial or financial relationships that could be construed as a potential conflict of interest.
